# Does correction of reverse shoulder arthroplasty angle improve clinical outcomes in cuff tear arthropathy?

**DOI:** 10.5152/j.aott.2021.21189

**Published:** 2021-11-01

**Authors:** Vahdet Uçan, Anıl Pulatkan, Murat Sarıkaş, Mehmet Kapıcıoğlu, Kerem Bilsel

**Affiliations:** Department of Orthopedics and Traumatology, Bezmialem Foundation University, İstanbul, Turkey

**Keywords:** Reverse shoulder arthroplasty, Reverse shoulder arthroplasty angle, Cuff tear arthropathy, Glenoid inclination, Asymmetrical reaming

## Abstract

**Objective:**

The aim of this study was to investigate the impact of correction of the reverse shoulder arthroplasty (RSA) angle on clinical outcomes in patients with cuff tear arthropathy (CTA).

**Methods:**

This single-center retrospective study was conducted in patients with CTA treated with RSA between 2013 and 2018. A structured questionnaire collecting demographic data, postoperative follow-up time, pre- and postoperative range of motion (ROM), American Shoulder and Elbow Surgeons (ASES) and Constant functional scores as well as scapular notching according to the Sirveaux Classification and RSA angle were evaluated by independent observers. The association between functional outcomes and RSA angle was analyzed using a curve estimation approach.

**Results:**

Seventy-four patients with a mean age of 69.4 ± 8 years and mean follow-up period of 38.2 ± 10.8 months were included the study. The medialized inlay component was implanted in 35 patients, and the lateralized onlay component was used in 39 patients. The mean preoperative ASES and Constant scores improved from 28.4 ± 5.1 and 31.1 ± 5.9 to 73.4 ± 23.3 and 70.5 ± 16, respectively, at the last follow-up (both *P* < 0.001). The mean pre- and postoperative RSA angles were measured to be 21.3 ± 9.3° and 5.5 ± 10.1°, respectively, on X-ray. The postoperative RSA angle was 10.4 ± 10.3° in computerized tomography (CT) scans. There was an excellent correlation between X-ray and CT measurements (rs: 0.971, *P* < 0.001). It was found that patients with good postoperative functional scores tended to have an RSA angle of 0-10° postoperatively. The delta internal rotation of the medialized design group was greater than that of the lateralized design group (*P* = 0.029).

**Conclusion:**

In patients undergoing RSA for CTA, satisfactory clinical outcomes can be obtained by achieving a postoperative RSA angle of 0-10° with an asymmetrical inferior reaming technique.

**Level of Evidence:**

Level III, Therapeutic Study

## Introduction

Reverse shoulder arthroplasty (RSA), first introduced by Neer in the 1970s and improved by Grammont in the 1980s, is now widely performed.^1^ While the main indication for RSA has been cuff tear arthropathy (CTA), indications have expanded to include massive irreparable cuff tears, proximal humerus fractures in the elderly, failed arthroplasty, and malignancy.^2,3^

Although advancements in prosthetic design have reduced complications, surgical technique plays an extremely important role. In particular, complications such as instability, glenoid loosening, scapular notching, and restricted range of motion (ROM) that are caused by suboptimal positioning of the prosthesis decrease patient satisfaction.^4^ Therefore, it is critical to fix the baseplate at least at a neutral inclination on the lower part of the glenoid surface to provide optimal deltoid tension and avoid inferior scapular impingement.^5-^^8^ One parameter recently specified for optimal implantation positioning of the glenoid component is the RSA angle.^9^

The primary aim of this study was to investigate the impact of RSA angle correction on clinical outcomes in patients undergoing RSA. The secondary aim was to compare the medialized inlay and lateralized onlay prosthesis designs on functional outcomes. The study hypothesis was that correction of the RSA angle via the asymmetrical inferior reaming technique would improve functional and radiologic outcomes.

## Materials and Methods

### Study design

This single-center, retrospective clinical study was conducted in Bezmialem Foundation University, Turkey, between 2013 and 2018. Local Institutional Review Board approval was obtained before starting the study (27.05.2020/1). Detailed information about the surgical interventions was provided to all patients, and each patient signed an informed consent form including information on the treatment alternatives, operative technique, and complications. The hospital data, patient records, and operative reports were reviewed to confirm age, sex, clinical examination, pre- and postoperative imaging, diagnosis, date of operation, intraoperative findings, and functional outcomes.

### Patient selection

Hospital data from 245 patients who underwent RSA were reviewed. Those who were followed up for less than 2 years (n = 40), who underwent surgery for an etiology other than CTA, such as proximal humerus fracture (n = 32), massive cuff tear (n = 30), hemiarthroplasty revision (n = 5), osteosynthesis failure (n = 10), malignancy (n = 3), or primary osteoarthritis (n = 25), and patients without sufficient documentation (n = 26) were excluded. The remaining 74 CTA patients who had at least two years of follow-up and sufficient documentation were included in the study.

### Clinical assessment

An orthopedic surgeon and physiotherapist who were blinded to the treatment modality and radiologic outcome performed the clinical assessments. Pre- and postoperative final follow-up active ROM, including forward flexion, abduction, external rotation at 90 degrees abduction, and internal rotation at neutral position, was measured using a universal goniometer and recorded as a degree. The Constant Score^10^ and American Shoulder and Elbow Surgeons (ASES) score^11^ were utilized for functional evaluation of the patients. The difference in functional outcomes from the preoperative period to the postoperative final follow-up was recorded as a delta value.

### Radiologic assessment

A senior musculoskeletal radiologist blinded to the treatment method and clinical outcome performed the radiologic evaluations. All patients underwent preoperative true shoulder anteroposterior X-rays, which allow better evaluation of joint congruency and the glenohumeral cartilage space.

**Figure 1.**
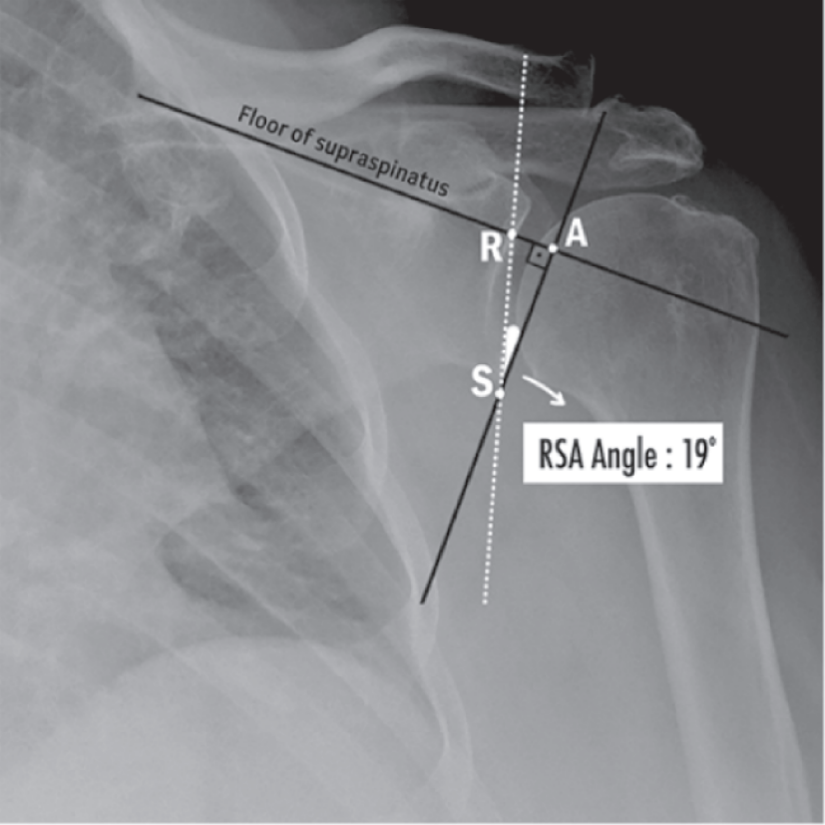
Preoperative X-ray demonstrate measurement of the RSA angle in X-ray. RSA, Reverse shoulder arthroplasty.

**Figure 2.**
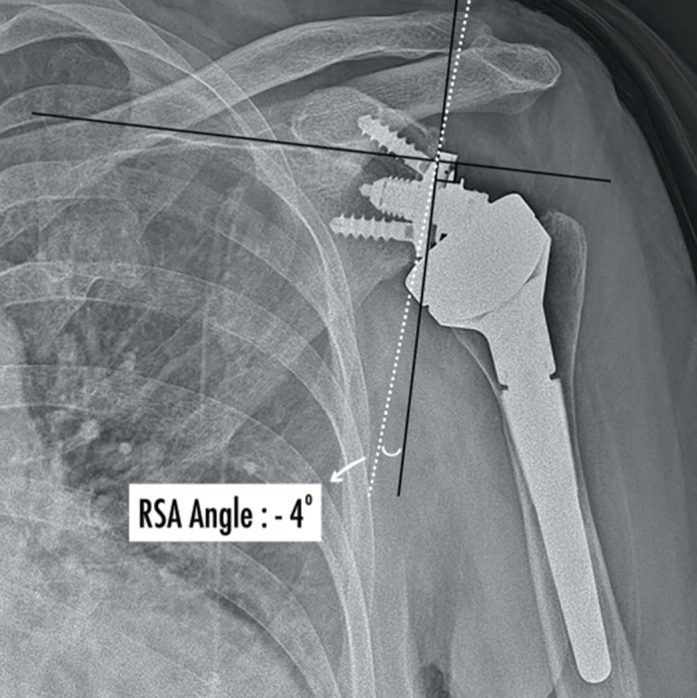
Postoperative X-ray demonstrate measurement of the RSA angle in medialized inlay prosthesis. RSA, Reverse shoulder arthroplasty.

**Figure 3.**
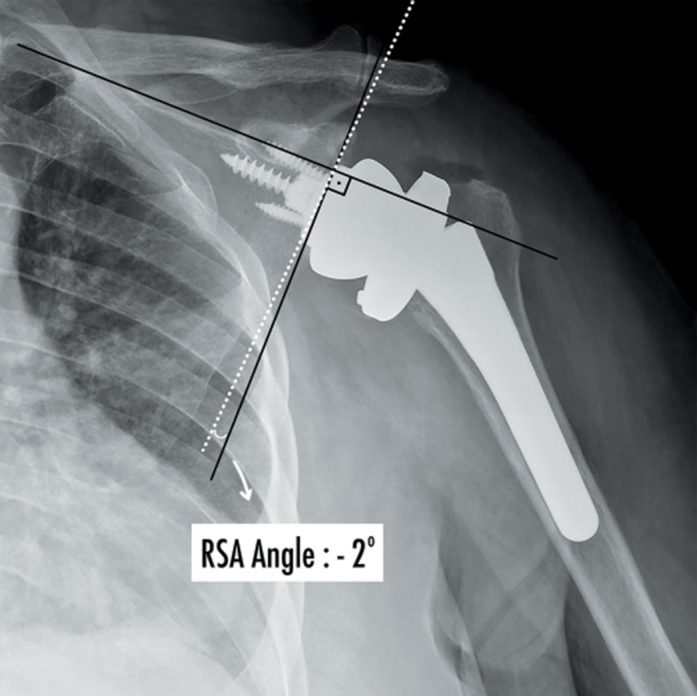
Postoperative X-ray demonstrate measurement of the RSA angle in lateralized onlay prosthesis. RSA, Reverse shoulder arthroplasty.

The RSA angle, defined as the angle between the inferior part of the glenoid fossa and the perpendicular to the floor of the supraspinatus, was measured in pre- and postoperative X-rays and postoperative computerized tomography (CT) scans on a commercial PACS workstation (Synapse PACS; Fujifilm Medical Systems, Stamford, CT) (Figure 1-3). In the measurement of the RSA angle, the R, S, and A points are determined separately. The intersection of the supraspinatus fossa line with the glenoid surface is shown by point R, the inferior edge of the glenoid is shown by point S, and the vertex of the right triangle created by the line of the supraspinatus fossa and a perpendicular line passing through point S is shown by point A; the *RS* line (lower surface of the glenoid) is the hypotenuse of the right triangle. The correlation between the measurements on X-ray and CT scan was evaluated. Scapular notching was also assessed in the final follow-up X-ray according to the Sirveaux Classification.^12^

### Surgical technique

All procedures were performed by a single senior shoulder surgeon (KB) with 10 years of experience in shoulder arthroplasty. All patients were operated on under general anesthesia. The patients were placed in beach chair position, and the skin was disinfected with 10% povidone-iodine before draping. For surgical prophylaxis, 2 g cefazolin sodium was administered 30 minutes prior to incision.

All patients were operated on using the same surgical technique. The prosthesis was placed through a deltopectoral incision with an asymmetrical inferior reaming technique in all patients. The upper 1 cm of the pectoralis major tendon was released. The biceps was tenotomized, and soft tissue tenodesis was performed. The peeling technique was preferred for mobilization of the subscapularis. The humeral head was dislocated and cut at a 20° retroversion angle with a 135°guide. The middle and inferior glenohumeral ligament and capsule were released, and the entire labrum was resected. The humerus was retracted posteriorly using a humeral head retractor. The long head of the triceps muscle was released from the bone margin using a scalpel in order to properly expose the inferior glenoid. Using an electrocautery probe, a circle was drawn in the lower part of the glenoid, and its center was marked. A guide pin was implanted at the marked point by aiming it with the proper version and inferior inclination to correct the preoperative RSA angle. The glenoid reaming was performed in periodically, not more than 1 cm at a time, until the subchondral bone was exposed. All baseplates were implanted on the inferior margin of the glenoid rim without an augmented baseplate or bone graft. A cementless humeral stem was inserted in each patient with 20° of retroversion. The baseplate was fixed with screws to the glenoid according to the type of prosthesis, and then the glenosphere was inserted onto the baseplate. The humerus was reduced, and the subscapularis tendon, if reparable, was repaired using a transosseous approach.

Two different prosthesis designs were used: medialized inlay (Systema Multiplana Randelli; LIMA, Udine, Italy) and lateralized onlay (Biomet Comprehensive Reverse Shoulder System; Warsaw, Indiana). The selection of prosthesis was made according to the surgeon’s judgement and the availability of products and services. Lateralization through the baseplate or glenosphere was not applied in any patient.

### Follow-up protocol

All patients used an arm sling with a 30° shoulder abduction pillow in neutral rotation for 4 weeks after surgery to preserve the subscapularis repair. Each patient was discharged from the hospital within 2 days after the operation with a home rehabilitation program that included exercises for deltoid function enhancement. Pendulum exercises were started 2 weeks postoperatively. Active external rotation was not permitted for 1 month. Active assisted exercises were started after the first month to achieve full ROM between the second and third months. Routine follow-up was performed at 2 weeks, 6 weeks, 3 months, 6 months, and 1 year and then annually thereafter.

### Statistical analyses

All statistical analysis was performed using the Statistical Package for Social Sciences (SPSS) statistical software package (IBM SPSS Corp., Armonk, NY, USA). Continuous variables were expressed using median (minimum-maximum) and mean ± standard deviation values, and categorical variables were expressed using frequency (percentage) values. Normality of the continuous data was tested using the Shapiro–Wilk test. Two-group comparisons were performed using the Mann–Whitney *U*-test. Categorical comparisons were performed using the Chi-square test. Relationships between nonnormally distributed variables were analyzed using Spearman correlation coefficient. To compare improvements in functional scores and ROM between groups, the analysis of covariance was used, with preoperative values as covariates. The association between related functional outcomes and RSA angle was analyzed using a curve estimation approach. The results and associated *P* values are reported. *P* values of <0.05 were considered to be significant.

## Results

### Patient demographics

All patients underwent unilateral RSA. The mean age of the patients was 69.4 ± 8 years. Regarding gender, 79.7% (n = 59) of the patients were female and 20.3% (n = 15) were male. The mean follow-up period was 38.2 ± 10.8 (range, 24-76) months.

### Functional outcomes

ROM increased significantly in patients after surgery, with forward flexion increasing from 56.8 ± 11.1° to 118 ± 15° (*P* < 0.001), abduction increasing from 35.6 ± 5° to 108 ± 15.8° (*P* < 0.001), internal rotation increasing from 13.8 ± 3.8° to 34.7 ± 6.4° (*P* < 0.001), and external rotation increasing from 18.3 ± 5.1° to 42.8 ± 9.6° (*P* < 0.001).

The average preoperative ASES and Constant scores improved at the last follow-up from 28.4 ± 5.1 and 31.1 ± 5.9 to 73.4 ± 23.3 and 70.5 ± 16, respectively (both *P* < 0.001).

### Radiologic outcomes

**Figure 4.**
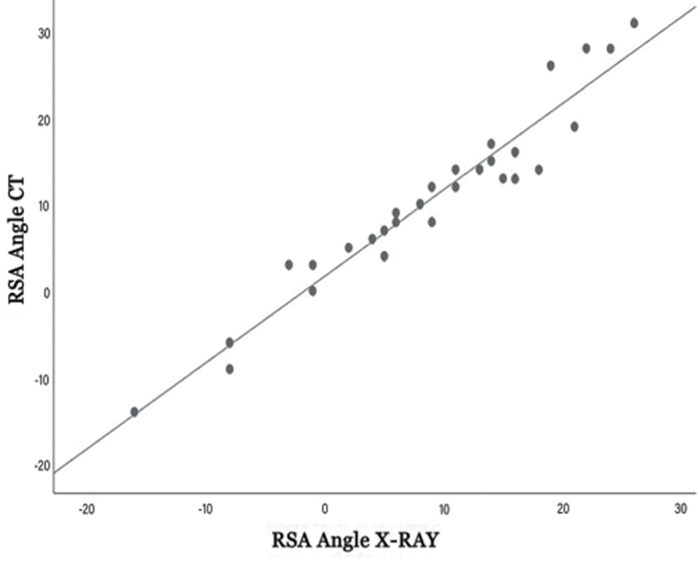
Correlation of the RSA angle measurement in postoperative X-ray and CT scan. RSA, Reverse shoulder arthroplasty.

On X-ray, the average pre- and postoperative RSA angles were measured to be 21.3 ± 9.3°and 5.5 ± 10.1°, respectively. Postoperative CT scan was performed in 30 of 74 patients (41%) for various clinical reasons. The average postoperative RSA angle measured by CT scan was 10.4 ± 10.3°. There was an excellent correlation between X-ray and CT measurements (rs: 0.971, *P* < 0.001) (Figure 4).

Scapular notching according to the Sirveaux Classification was grade 0 in 39 (53%) patients, grade 1 in 32 (43%) patients, and grade 2 in 3 (4%) patients. The RSA angles of patients with scapular notching grades 0, 1, and 2 were 3.8 ± 9.6, 6.1 ± 10, and 21.3 ± 5, respectively. There was no significant difference between grades 0 and 1 in terms of RSA angle (*P* = 0.298). Although the mean RSA angle of patients with grade 2 scapular notching was higher than that of patients with other grades, the patients in this group could not be compared with those with other grades due to the small sample size.

### Association between functional outcomes and RSA angle

**Figure 5.**
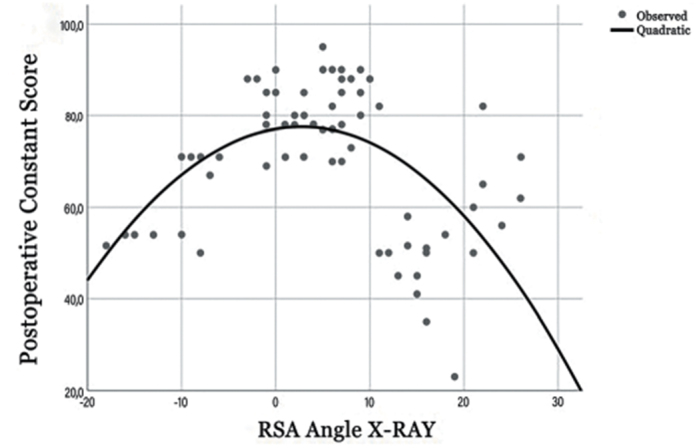
The model for RSA angle in X-ray and the postoperative Constant functional score.

**Figure 6.**
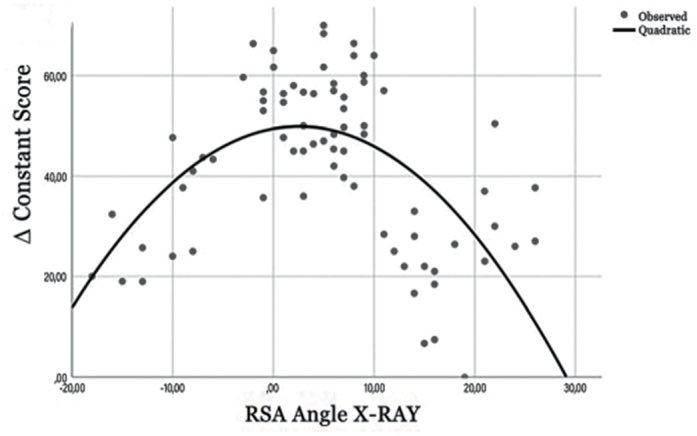
The model for RSA angle in X-ray and the delta Constant functional score.

**Figure 7.**
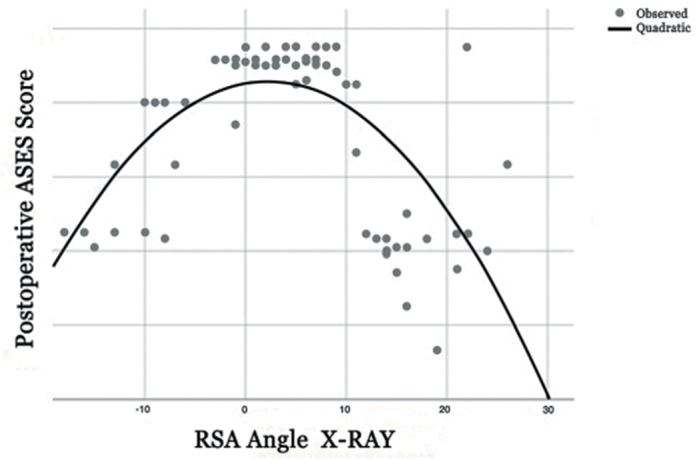
The model for RSA angle in X-ray and the postoperative ASES functional score.

**Figure 8.**
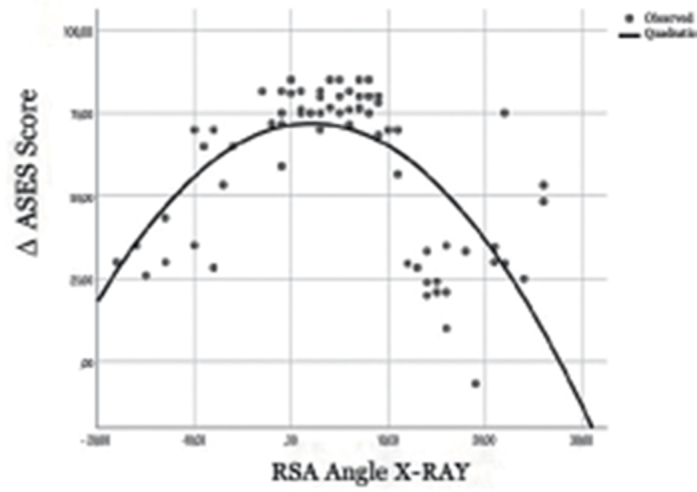
The model for RSA angle in X-ray and the delta ASES functional score.

The most appropriate curve estimation model in terms of coefficient significance, model significance, and the determination coefficient for functional outcomes and RSA angle was the quadratic model. This model showed that functional outcomes (postoperative forward flexion, delta forward flexion, postoperative abduction, postoperative internal rotation, delta internal rotation, postoperative external rotation, postoperative ASES score, delta ASES score, postoperative Constant score, delta Constant score) tend to improve in patients with a postoperative RSA angle of 0-10° (Figure 5-8 and Table 1).

### Prosthesis design

Medialized inlay design prostheses were implanted in 35 patients, and lateralized onlay design prostheses were implanted in 39 patients. In patients with similar demographics, the delta internal rotation in the medialized design group was greater than in the lateralized designs (*P* = 0.029) (Tables 2 and 3). Scapular notching was observed in 17 patients in the medialized design group and 18 patients in the lateralized design group. There was no significant difference in terms of scapular notching between the two groups (*P* = 0.835). In both groups, there were two complications without significant difference (*P* = 0.911).

### Complications

Four patients (5%) suffered complications after surgery. In one of these, prolonged wound drainage developed due to a superficial surgical site infection. This was treated with irrigation and surgical debridement during the second postoperative week, and the patient remained free of symptoms. Neuromuscular electrical stimulation was used for a patient who suffered postoperative anterior deltoid paresis, and the patient’s symptoms regressed. Hematomas occurring in 2 patients were treated conservatively. Periprosthetic fracture, instability, and glenoid or humeral component loosening were not observed in any patient.

## Discussion

The significant findings in this study were that functional outcomes tend to improve in patients undergoing RSA for CTA with a postoperative RSA angle of 0-10° and that correction of RSA angle can be achieved with the asymmetrical inferior reaming technique. There was no significant difference between the X-ray and CT scan RSA angle measurements. In addition, no clinically significant difference was found between the medialized inlay and lateralized onlay groups in terms of functional outcomes.

The vectors of the remaining cuff muscles are orthogonal, and potentially more efficient, and functional outcomes are satisfactory when the postoperative RSA angle is 0° (neutral inclination).^11,13,14^ However, it has been stated that to achieve a postoperative RSA angle of 0°, a large amount of bone must be reamed from the inferior part of the glenoid, causing bone stock loss and excessive medialization of the baseplate. Dilissio et al.^5^ reported that 0° inclination cannot be achieved with surface referencing or subchondral smile techniques and that excessive medialization to overcome this situation may result in prosthesis instability, scapular notching, glenoid loosening, and decreased ROM. An augmented baseplate or inferiorly inclined bone graft can be used to avoid these complications. However, no clinically significant difference in terms of functional outcomes was shown in several studies comparing standard RSA with lateralized offset RSA through baseplate.^15–18^ In our study, patients’ functional outcomes tended to improve at postoperative RSA values of 0-10°, a range that includes our average postoperative RSA angle. Furthermore, our functional outcomes (ASES score: 73.4 ± 23.3; Constant score: 70.5 ± 16) were satisfactory when compared with the current literature. The RSA angle resulting in optimum outcome following RSA may vary depending on the surgical technique. There is a need for studies investigating the impact of the RSA angle on functional outcomes following different surgical techniques.

As functional outcomes are known to be satisfactory when the glenoid baseplate is positioned correctly, preoperative radiologic planning should be performed.^19–23^ Boileau et al.^9^ argued that the angles that have been used to date for planning the glenoid placement are insufficient to achieve 0° baseplate inclination. They also reported that the entire glenoid surface should not be measured for the component, which is placed in the inferior part of the glenoid, and defined the RSA angle, which indicates the inclination of the inferior part of the glenoid. In their study of 47 patients, the mean RSA angle was 25 ± 8° on preoperative X-rays, 20 ± 6° on 2D reformatted CT scans, and 21 ± 5° on 3D CT reconstructions. With the 3D CT measurements considered to represent the gold standard, the accuracies of X-ray and 2D CT were found to be 67% and 82%, respectively.^9^ Duethman et al.^24^ reported that the average preoperative RSA angle was 33 ± 11.2° and the average postoperative RSA angle was 6.6 ± 9° in 147 patients who underwent RSA and were followed for at least 5 years. They concluded that scapular notching was correlated with higher RSA angles. In our study, the average preoperative RSA angle was 21.3 ± 9.3° on X-ray. The average postoperative RSA angle was 5.5 ± 10.1° on X-ray and 10.4 ± 10.3° on CT scan, respectively. An excellent correlation was found between X-ray and CT measurements of 30 patients with postoperative 2D CT scan (rs: 0.971, *P* < 0.001). We are of the opinion that X-ray evaluation is sufficient to evaluate the postoperative RSA angle. Regarding the association between the RSA angle and scapular notching, the RSA angles of patients with scapular notching grades 0, 1, and 2 were measured to be 3.8 ± 9.6, 6.1 ± 10, and 21.3 ± 5, respectively. Although the mean postoperative RSA angle in patients with Grade 2 scapular notching was significantly higher than that in the other groups, statistical comparisons could not be made due to the insufficient sample size.

Patients who undergo RSA for CTA have improved functional outcomes regardless of prosthesis design; however, it is unclear if one design is superior.^25–27^ In a systematic review of 18 studies, Helmkamp et al.^28^ reported an average improvement of 54° in forward flexion, 62° in abduction, and 21° in external rotation in the lateralized design group, versus improvements of 60°, 55°, and 7°, respectively, in the medialized design group. They concluded that the lateralized design prosthesis showed significantly increased postoperative external rotation and decreased scapular notching. In a recent review, Parry et al.^29^ theorized that humeral lateral design prosthesis may be useful for improving the mechanics of the remaining rotator cuff and deltoid musculature and concluded that functional outcomes with lateralized prostheses are improved compared to those with medialized prosthesis. In our study, the onlay design lateralized from humerus with 147° metaphyseal inclination and Grammont type design medialized from humerus with 150° inclination were compared. In addition, the lateralized glenoid component with offset was not applied in any patient. No significant difference was found between the groups in terms of outcome scores. The medialized group had superior improvements in internal rotation, but this was not clinically significant (18.3 ± 7.1° vs 14.7 ± 6.9°).

This study has several limitations, including its retrospective approach and the small number of patients. Radiologic measurements were performed by only one senior musculoskeletal radiologist, so the inter- and intraobserver reliability could not be calculated. Another limitation of the study is the absence of quantification of loss of muscle strength. The fact that only one senior surgeon performed the operations could be considered a limitation. Another limitation is that the impact of the RSA angle on functional outcomes using the asymmetrical inferior reaming technique was not compared with the outcomes of other surgical techniques, such as a metallic superior augmented baseplate or an inferiorly inclined bone graft. Although the standard designs of both arthroplasty options with lateralized and medialized humeral component without any offset from the glenoid component were compared, there are multiple different modifications of these arthroplasty designs, which can be compared with high number of patients. While all patients included in the study had CTA, another limitation is that glenoid defects were not evaluated and classified before surgery.

Functional outcomes after RSA using the asymmetrical inferior reaming technique for CTA tend to be improved in patients who have a postoperative RSA angle of 0-10°. Therefore, a postoperative RSA angle of 0-10**°** should be targeted via preoperative radiologic planning. Further prospective randomized controlled comparative studies enrolling more patients and with longer follow-up are needed to investigate the impact of correction of the RSA angle using different surgical techniques on functional outcomes in patients undergoing RSA for CTA.

**Highlights**

- In reverse shoulder arthroplasty, optimal implantation of the glenoid component is crucial.
- The “reverse shoulder arthroplasty angle” is one of the new parameters that help surgeons to place the glenoid component in an optimal position.
- Functional outcomes after reverse shoulder arthroplasty using the asymmetrical inferior reaming technique for cuff tear arthropathy tend to be improved in patients who have a postoperative RSA angle of 0-10°.

## Figures and Tables

**Table 1.** Univariate Quadratic Modeling of Functional Outcomes Based on RSA Angle

	Functional Outcome											
	Forward Flexion		Abduction		Internal Rotation		External Rotation		ASES Score		Constant Score	
	Postop.	Delta	Postop.	Delta	Postop.	Delta	Postop.	Delta	Postop.	Delta	Postop.	Delta
F	17.149	14.3	18.533	0.435	11.247	11.799	5.156	2.212	28.971	27.350	16.967	19.784
*P*	**< 0.001**	**< 0.001**	**< 0.001**	0.649	**< 0.001**	**<0.001**	**0.008**	0.117	**<0.001**	**<0.001**	**<0.001**	**<0.001**
R^2^	0.326	0.287	0.343	0.012	0.241	0.249	0.127	0.059	0.449	0.435	0.323	0.358

ASES, American Shoulder and Elbow Surgeons; Postop., postoperative. (Bold values denote statistical significance at the p < 0.05 level.)

**Table 2.** The Comparison of Patient Demographics between Medialized Inlay and Lateralized Onlay Prostheses

	Medialized Design (n = 35)	Lateralized Design (n = 39)	*P* Values
Age (years)	68.9 ± 7.9	69.8 ± 8.1	0.661^a^
Gender (female/male)	26/9	33/6	0.27^b^
Follow-up (months)	40.5 ± 12.3	36.2 ± 8.9	0.164^a^
Preoperative RSA angle (°)	20.7 ± 8.2	22.3 ± 10.3	0.497^a^
Postoperative RSA angle (°)	4.7 ± 8.6	6.2 ± 11.4	0.432^a^

a Mann–Whitney *U*-test.

b Chi-Square test.

**Table 3.** The Comparison of Patient Functional Outcomes between Medialized Inlay and Lateralized Onlay Prostheses

	Medialized Design (n = 35)		Lateralized Design (n = 39)		*P* Values	
	Preoperative Score	Delta Score	Preoperative Score	Delta Score	Preoperative Score	Delta Score
Forward flexion	57.1 ± 11.5	84 ± 14.3	56.4 ± 10.9	81 ± 16.7	0.717^a^	0.427^b^
Abduction	35.4 ± 5.1	37.3 ± 7.6	35.8 ± 5.1	37.6 ± 7.1	0.851^a^	0.832^b^
Internal rotation	14.1 ± 3.7	18.3 ± 7.1	13.5 ± 3.8	14.7 ± 6.9	0.53^a^	**0.029** ^b^
External rotation	18.4 ± 5.1	25 ± 10.1	18.2 ± 5.2	24 ± 11.6	0.857^a^	0.875^b^
Constant score	31.1 ± 5.9	44.3 ± 15.3	31 ± 6	40.2 ± 17.8	0.056^a^	0.274^b^
ASES score	28.1 ± 5.2	65 ± 21.1	28.6 ± 5	54.8 ± 25.4	0.987^a^	0.067^b^

a Mann–Whitney *U*-test.

b Analysis of covariance (ANCOVA).
